# Prevention of Disasters Related to Extreme Natural Ground Deformation Events by Applying Spatial Modeling in Urban Areas (Quito, Ecuador)

**DOI:** 10.3390/ijerph17030753

**Published:** 2020-01-24

**Authors:** Marcelo Cando-Jácome, Antonio Martínez-Graña, Virginia Valdés

**Affiliations:** Geology Department, External Geodynamics Area, Faculty of Sciences, University of Salamanca, Plaza Merced s/n, 37008 Salamanca, Spain; id00709713@usal.es (M.C.-J.); vvaldes@usal.es (V.V.)

**Keywords:** natural disasters, spatial modeling, ground deformation, INSAR, exposure urban

## Abstract

Synthetic Aperture Radar Interferometry (InSAR) is a spatial technique based on obtaining the phase differences of two radar images, acquired by a satellite from separate orbits and at different times, to obtain a ground displacement image of a study area, This image is called interferogram. On the other hand, space syntax is a technique within architecture that is applied to quantify and describe the level of ease of population movement through any urban space in a city. It analyzes the flow, transit, displacement, accessibility and concentration of the population in areas of basic services, health, security, commerce and entertainment. What would happen if an earthquake greater than 6 or 7 Moment Magnitude-Mw occurs in these areas of intense concentration of the population that are in buildings constructed on intense deformations of the land? With respect to the seismic risk in the city of Quito, many studies related to seismic risks have been published, but there are no studies that relate the deformation of the land (INSAR) with the space syntax, so this article presents a new vision in the joint application of these tools, a useful vision for urban planners and designers, considering the occurrence of a major earthquake in areas of buildings that are located on intense land deformations and have high population concentrations. This study has been prepared in two phases: in the first phase, the built-up areas concentrated in the greatest terrain deformations by accumulated displacement obtained using the APS estimation & multitemporal analysis by PSI-InSAR time series analysis methodology and Sentinel 1A and 1B satellite images were categorized. In the second phase, through the space syntax’s theory and the use of DepthmapX, the movement patterns and traffic flows of the population were determined by means of graphs of spaces interconnected by streets (axial maps), to predict the spatial behavior of humans and its concentration in the mentioned sites. Finally, the results were integrated, determining the degree of exposure of the population found in built areas with high to very high displacement and an intense population concentration.

## 1. Introduction

The criterion of this study is from the point of view of the forecast, which means “having exposure maps to natural hazards” before they are activated and cause disasters such as the 7.8 Seismic Moment Magnitude-Mw earthquake, which occurred on the Ecuadorian coast on the 16th. April 2015, that in the area of greatest destruction of the city of Manta, the collapse of the Felipe Navarrete Shopping Center that was located in an intense land deformation zone caused more than 96 deaths.

Historically, the seismic risk of buildings has been analyzed by traditional methods like direct methods (by type of construction, mechanics); indirect methods (vulnerability index, seismic intensity, rapid selection from ATC-21); conventional methods (heuristics, vulnerability index) and hybrid methods (combine the characteristics of the previous methods) [1]. The use of the INSAR methodology to analyze seismic risk also exists in much scientific literature.

No scientific research has been found that combines the INSAR methodology with the Syntax of Space to analyze the exposure of engineering constructions located in areas of deformation of the land and their relationship with the transit-concentration of the population in the event of an intense earthquake. There is not much scientific literature that mentions the use of space syntax and natural disasters such as earthquakes, floods and tsunamis. According to Stonor’s presentation at the First Conference on Space Syntax, held in China, Beijing, 5 December, 2015 [2], the analysis of spatial syntax is increasingly used to advise government agencies on the impacts of devastating events, whether created by man or created by nature.

Before the earthquakes, the analysis showed that the central area had a strong “multi-scale” space core of pedestrian concentration. This means that the central area was simultaneously important for local and global population movement scales, a typical finding for central commercial areas, as they thrive by accessing multiple movement scales: people walking towards them locally and people driving or by bicycle as part of your transit larger trips. After the earthquakes the spatial hierarchy shifted with some important consequences. First, several local, peripheral centres became significantly more important. Many businesses relocated there from the city centre. Second, some new centres emerged when the spatial configuration shifted sufficiently to make these more strongly connected within the remaining network of the city than they had been previously. The centre on Stanmore road is one such example of a zone where businesses relocated to.

Other examples of disaster analysis include spatial modelling of the effects of flooding and terrorism incidents, when the spatial layout of an urban centre may be disrupted for hours, days or longer and when emergency services, as well as the general public, need to understand how the hierarchy of space has been affected so that they can replan their activities.

Finally, the future will see the construction of very large spatial network models in order to produce national and regional spatial strategies that are informed by space syntax analysis—bringing a new level of rigour to such activities.

This innovative study is considered because it is the first time it is used in the country as a forecast mechanism. The aim of the combination INSAR with the syntax of space to analyze the exposure of buildings located in areas of intense land deformation and with a high concentration of the population that can collapse, to predict economic and human life losses in the event of an intense earthquake such as the one that occurred in the city of Manta on the Ecuadorian coast, where an earthquake of 7.8 Mw caused the collapse of several buildings including the Felipe Navarrete shopping center.

According to world statistics, about three quarters of all earthquake-related deaths are due to the collapse of buildings [3]. In Ecuador this was evidenced in the 7.8 Seismic Moment Magnitude-Mw earthquake of April 16, 2015, with an epicenter in Pedernales city on the Ecuadorian coast. More than 96 people died due to the collapse of the Felipe Navarrete Shopping Center located in the zero zone of Manta city. This shopping center was located on an intense ground deformation area, which was of tectonic origin [4].

To avoid the possibility that an earthquake greater than 6 Mw can cause victims and destruction of the strategic infrastructure in the La Mariscal area, located in the central-east area of the city of Quito, in this study, a methodological proposal is presented with the objective of zoning the seismic exposure of buildings existing in this part of the city in three phases. In the first phase, the deformation and the accumulated displacement of the ground (total displacement value in mm of a ground point in reference to a relative starting point or known stable point measured in a series of times) were determined with atmospheric phase screen (APS) and doing PS-InSAR time series analysis methodology, based on Sentinel 1A and B images, in a period of time from 2 September 2015 to 28 November 2019. In the second phase, the concepts and tools of space syntax and the use of DepthmapX were applied, to determine the movement patterns and traffic flows of the population through graphs of spaces interconnected by streets and their intersections (axial maps), to predict human spatial behavior and its concentration in places of intense commerce, entertainment and public services. Finally, the results were integrated, determining the exposure intensity of the population living in built areas with high to very high displacement and a high concentration of the population. This study has focused on exposure from the point of view of the susceptibility of buildings and the population to be affected by a specific seismic hazard and the probability of being exposed to intense deformations of the relief that can move with earthquakes of more than 6 or 7 Mw [5].

The city of Quito is exposed to a high seismic risk [6]. The capital has been affected by earthquakes occurring beneath it, in the Quito fault, and by large earthquakes that occurred in the Inter-Andean valley or in nearby mountain ranges. The last major earthquake that affected Quito was in 1868. At that time, the population of the city consisted of some 45,000 people living in an area of 4 km^2^ [7]. Since that date, a rapid growth of the city has been evidenced, which gave way to a large number of low quality buildings and edifications in unstable sites such as ravines filled with loose soil and steep slopes, and the increase in population to more than two and a half million inhabitants, so if a large earthquake were to occur, the damage would be considerable [6]. Current seismicity studies show that every 50 years (on average), there have been earthquakes with epicenters located at distances close to the capital city, which have caused significant damage to it. As of 1541, at which time historical records started, seven earthquakes with intensity VI or higher were felt in the capital [8]. This intensity value of the Medvédev-Sponheuer-Kárník-MSK intensities scale corresponds to an earthquake in which people became frightened and left their homes, and the old mud, adobe or masonry walls were severely damaged, with the occurrence of small landslides. The two closest earthquakes that affected the city of Quito with greater intensity were that of Guayllabamba (6.4 Mw in 1587) and that of Quito (7.2 Mw in 1859). Both had an intensity of VIII of the MSK scale which according to reports of the time, caused difficulties for residents to stand and the collapse of old buildings occurred. In addition to the mentioned earthquakes, other distant earthquakes have caused damage to the city. Among them can be mentioned the ones of Ambato (7.6 Mw, 1698) and Riobamba (7.6 Mw, 1797) to the south of the capital city; Ibarra (7.2 Mw, 1868) to the north and Baeza (6.9 Mw, 1987) to the east of the city.

In recent times, and after the mega earthquake of 27 February 2010 in Chile, which had a magnitude of 8.8 and left a death toll of more than 500 citizens with economic losses estimated at 30 billion dollars [6], the authorities of the Ecuadorian government, focused on the reduction of seismic vulnerability in major cities located in the Ecuadorian coastal profile in the event of an earthquake of that magnitude, taking into account that in 1906 there was an earthquake of 8.8 on the coast of Esmeraldas province. According to specialized researchers [8], the losses caused by an event of this magnitude would duplicate or triplicate the ones of Chile. This approach involves the analysis of seismic vulnerability in inland cities located in the Highlands, Littoral Region and Galapagos Islands.

This concern in the Ecuadorian authorities led to the creation of the Ecuadorian Building Code of 2015 NEC-SE-DS, Seismic Loads and Resistant Earthquake Design, a code in which the elastic design spectrum (ground acceleration and displacement) was regulated.

**Figure 1:**
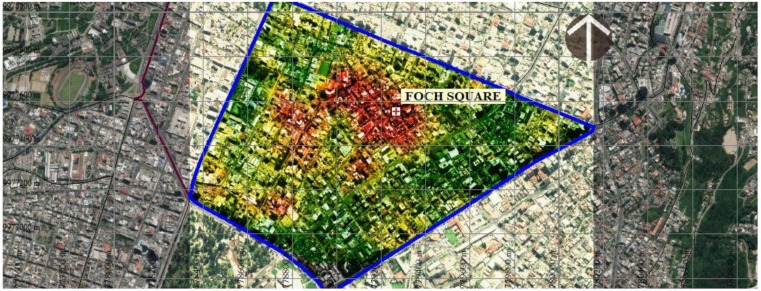
Concentration of economic activities in the La Mariscal neighborhood. The sectors with the largest agglomeration of productive economic activities (red). The sectors with the lowest agglomeration of these activities (green).

With respect to La Mariscal area, according to the Geophysical Institute of the National Polytechnic School (EPN), this sector of the city of Quito is built on an important seismic-tectonic zone with a system of geological faults of approximately 50 km in length that crosses the city. This means that La Mariscal area is vulnerable to the occurrence of an earthquake greater than 6 Mw that can cause damage to the population and its public and private infrastructure as well as to the environment and the economy of the sector [9]. In this sector there were four earthquakes of 6 and 7 Mw indicated within the green rectangle of Figure 1.

In addition to the loss of human lives, earthquakes cause serious impacts on residential areas and urban systems, including disruptions in the operations of the urban structure, damaging streets and roads that affect connectivity and accessibility to different areas and disable the normal use of the city. In this context, the urban structure of the city before and after an earthquake provides an important basis for the city’s activities to continue to function in a minimal state and play a key role in the resistance of the urban system to such hazards [10]. Understanding what the state of previous seismic susceptibility is and how an earthquake affects the behavior of the city and its urban structure, becomes a basis for territorial planning, the design of strategies to reduce this hazard and plan the actions of immediate response during and after the earthquake.

From the point of view of space syntax, the built urban space structure is closely related to the operation of the city and the use given by its inhabitants [11]. There is a strong correlation between the spatial patterns of human movement and the socioeconomic processes that have an impact on the urban spatial configuration. These patterns influence the use of the land [12] and the distribution of commercial and residential functional areas [13]. From this perspective, the analysis based on the spatial configuration as a mean to understand the operation of the city, represents an important tool to evaluate the impact on the patterns of the urban structure caused by an earthquake.

After an earthquake, the process of evacuation of the population must begin automatically and be autonomous [14], from the location of meeting points, escape routes and safe places that generally, in situations of chaos during evacuation, people face problems in making decisions regarding the search for these routes and as a result, evacuees tend to use any type of recognizable routes as possible evacuation paths that must be specified as safe [15].

Because the evacuation behavior of people has been closely associated with normal or routine movement patterns that are used daily [16], the concept of guidance that directs people through physical environments and improves their understanding of space, focuses on the person and their abilities to know their daily physical space, especially the streets where they travel and the places where they usually attend. This daily knowledge can be used to develop a signaling network in emergency situations, with the purpose of developing a network of possible safe evacuation routes for the population.

Several researchers have demonstrated through experimental tests that individual spatial decisions in signaling behavior are strongly affected by the spatial configuration of the built urban environment [17]. In this context, the theories of space syntax have tried to understand the way in which pedestrians move around their environment, showing through “natural movement” that the configuration of the street network is the main pattern generator of pedestrian movement to and from the buildings where they carry out their daily activities [18]. This argument becomes a fundamental issue when analyzing the behavior of people during the emergency and the selection of factors that influence their orientation from a spatial perspective in vulnerable areas [19].

Given the strong correlation between the population movement flows observed in the concurrence centers of the study area and those determined with the space syntax, this work suggests that spatial structures located in areas with high seismic exposure in this part of the city, must be analyzed before a destructive earthquake occurs through the relationship: deformation of the land - transit-concentration of the population, including the road network and open spaces (parks and green areas). This analysis allowed to identify the meeting points and streets with low or no exposed that people use daily and that may be the most likely routes to be selected as a safe evacuation to guide people to safe areas in case of emergency.

## 2. Materials and Methods

The Mariscal area of the city of Quito, capital of the Republic of Ecuador, has been selected for this study. This neighborhood is one of the areas with the highest seismic susceptibility of the city and is an area with high diversity of economic activities, with high levels of population agglomeration. This activity is especially characterized by the presence of business, centers, and is considered as “Zona Rosa”, where most of the bars, restaurants and entertainment sites are located. Figure 1 shows a graph presented by the Special Plan team “La Mariscal”-STHV-DMDU-2017 [20] in which the sectors with the largest agglomeration of productive economic activities represented in red are compared with the sectors of smaller agglomeration of these activities in green color. The concentration of establishments is located in economically strategic sectors or centralities. For example, the sector of Foch Square and its immediate environment that has the largest agglomeration of establishments, followed by Colon Avenue and the south-western administrative sector, close to Patria Avenue and August 10 Avenue.

This study does not focus the theories on atmospheric phase screen (APS) and PS-InSAR time series analysis methodology and spatial syntax analysis, but will briefly review its theoretical concepts. This study refers more to the results in the analysis of the surface deformation of the land (displacement of the land), the concentration of the urban spatial visibility of the constructed space network-population density and its exposure to the occurrence of an earthquake greater than 6 or 7 Mw.

The study in this part of the city was carried out to understand the role of the urban structure in relation to an earthquake before the seismic event occurs. In a first phase, soil deformation was analyzed using the atmospheric phase screen (APS) and PS-InSAR time series analysis methodology. In a second phase, the theory of space syntax was applied to understand the structure and functioning of La Mariscal before the earthquake, based on the analysis of spatial properties and the distribution of urban centers. To understand the syntax of space in socioeconomic development, this work focuses on determining the relationship between the population and the level of urban structural development of the neighborhood. In this case the level of urban structural development of La Mariscal, has a high degree since the population has at its disposal all municipal and private security, health, health, education, commerce and entertainment services, with buildings of reinforced concrete structures, with first class streets and avenues, which makes it more sustainable. This shows that the street network of this neighborhood and its accessibility, facilitates neighborhoods to have integrated socio-economic processes that lead to better environmental, social, economic and population welfare conditions. Unlike the peripheral districts of the city that are segregated and do not have integrated urban space structures and do not have basic sanitary, health, education, construction or trade services for their well-being.

**Figure 2:**
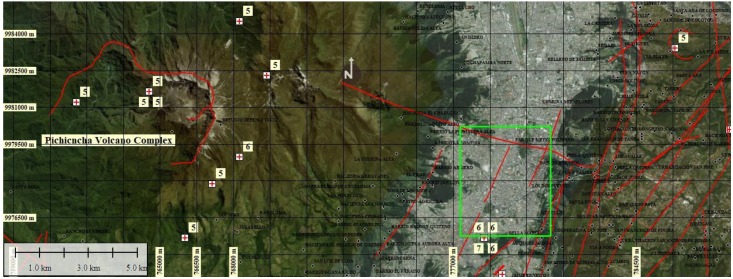
Location of La Mariscal in the Quito city (green polygon). Geological faults (red lines) and earthquake epicenters (red crosses). Base image source: Esri, DigitalGlobe, Google https://services.arcgisonline.com/ArcGIS/rest/services/World_Imagery/MapServer.

Finally, these two phases were related to spatially delimit the surface deformation of the soil (soil lifting-subsidence-accumulated displacement) with the density/flow-transit of the population. Figure 2 shows La Mariscal area that is located in the central part of the Quito city (green polygon) and its relationship with geological faults (red lines), more intense earthquake epicenters (red crosses) that influence the stability of this sector. In La Mariscal there have been four earthquakes of 6 and 7 Mw.

### 2.1. Phase 1: Interferometry of Synthetic Aperture Radar. Brief Summary of the Atmospheric Phase Screen (APS) Estimation & Multi-Temporal Analysis Methodology

**Figure 3:**
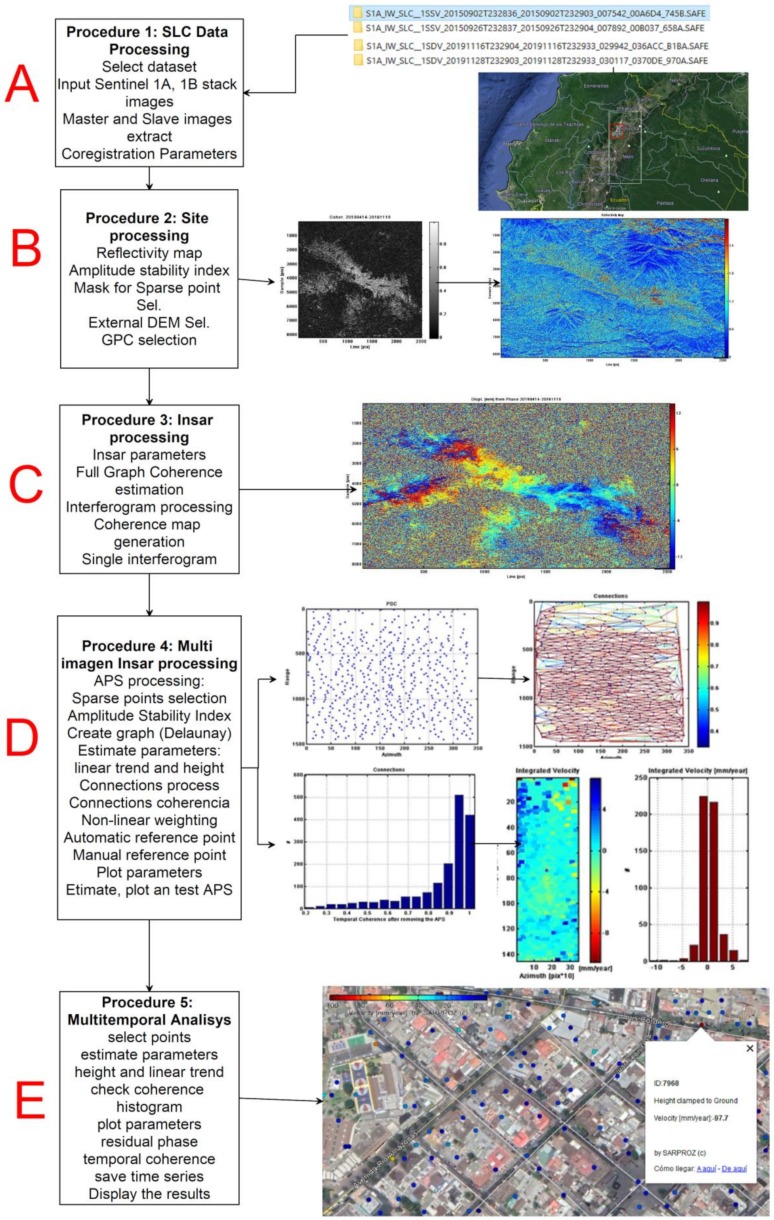
The SARPROZ software procedures used to obtain ground deformations by atmospheric phase screen (APS) estimation & multitemporal analysis. Description for (**A–E**) is given inside the figure.

The relief deformation and the accumulated displacement were performed with SARPROZ software [21,22], which has a wide range of tools for multi temporal–PS-INSAR processing. The flowchart of the procedures of this phase can be seen in Figure 3, which shows Sentinel 1A and 1B images obtained from the Alaska Satellite Facility [23]. These images correspond to a stack of 32 scenes with Level-1 single look complex (SLC)**,** from 2 September 2015 to 28 November 2019.

One of the main limitations in measuring ground deformation using synthetic aperture radar interferometry (InSAR) is atmospheric phase delay effects. In volcanic regions, as in this case, the atmospheric phase delay effects can cause serious problems in detecting volcanic unrest because atmospheric thickness is inversely related with the elevation of a volcanic mountain. It is commonly known that the atmospheric phase screen (APS) can be decomposed spatially into stratified and turbulent components.

In this study the APS to atmospheric noise reduction and persistent scatterer interferometry methodology is applied to time series analysis. SAR images, acquired by Sentinel 1 A and B, are used to derive ground displacement maps and deformation scenarios for La Mariscal area.

Regardless of the current occurrence of an intense earthquake, SAR images were used, covering the period between 2 September 2015 to 28 November 2019, to analyze using the PS-InSAR technique in a 32-cell stack. PS-InSAR images, the soil displacement characterized by long-term millimeter accuracy. Persistent scatterer PS-InSAR, also called coherent target monitoring (CTM), is an InSAR stack processing technique that uses multiple images taken at regular intervals to achieve improved measurement results. Stack processing allows surface movement to be determined by the temporal variation of phase for each pixel over time. The method focuses on stable and precise dispersers that do not suffer noise and provide a deterministic signal. These are the called persistent scatterers (PS) and they provide a stable phase history during the time period of image acquisition. The PS phases are stable over time and do not suffer from temporal decorrelation, allowing long-time observation and deformation monitoring.

**Figure 4:**
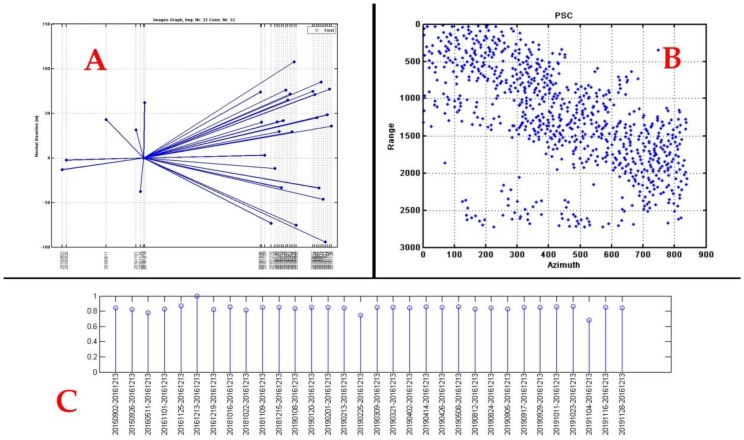
(**A**) Graph showing Sentinel 1 dataset analyzed in the normal baseline-temporal space, where each point and line represents an image and an interferogram, respectively. (**B**) Plot of sparse point selection. (**C**) The number of images in the stack with coherence greater or equal to 0.65.

Considering that the complete theory of this method is not the central theme of this document, the procedures followed are described:

*Procedure 1: Prepare the SLC Data Processing (A)*. This is a standard procedure to import or update data extracted from Sentinel 1 images. Select data set: import single look complex (SLC) Sentinel 1A-1B stack images. Set orbits to read and set the orbits of the scene. Master and slave images extraction; Select master image: the software can automatically select master images, but the master image can also be selected manually In the stars graph of Figure 4A, the master image is near the middle of the temporal and perpendicular baseline domain between Sentinel 1A images to the left and 1B to the right, to try to minimize the effects of normal and temporal baselines; Select slaves images. Coregistration parameters: with this procedure, it was possible to place the pixels of the master image with the slave images with the same corresponding pixels to perform interferograms correctly.

*Procedure 2: Site processing (B).* Preliminary anlalysis. Reflectivity map: with this function the software generates the reflectivity map as the temporal average of all images of the dataset that have been chosen to process. Amplitude stability index: is a single number which gives a statistical property of the amplitude series. Mask for sparse point selection: a threshold of the amplitude stability index value will be inputted, with values greater than the threshold to be selected and the remaining will be masked. Figure 4B. External DEM selection: used to remove the topographic phase and geocoding images. Ground control point (GPC) selection: used to orbital correction, geocoding, nterferogram flattening, persistent scatterers. Full graph coherence: used to estimates the coherence of all possible connections (interferograms) in the images space.

*Procedure 3: Insar processing (C).* Insar parameters: with this option, the parameters for INSAR processing are chosen. Full graph coherence estimation: used to read the interferograms according to loaded images graph and calculates the spatial coherence map as the average coherence of the set of current interferograms. Interferogram processing: used to processes interferograms according to the loaded images graph. An interferogram is generated between the master image and each of the slave images. Coherence map generation: used to read the interferograms according to loaded images graph and calculates the spatial coherence map as the average coherence of the set of current interferograms. Single interferogram: used to choose freely one interferogram to process and visualize any of the slave images between the master image.

*Procedure 4: Multi Imagen Insar processing (D).* Atmospheric phase scree (APS) estimation. Sparse points selection: used to select PS candidates (the reference point is generally placed at a location that is stable in terms of deformation), create a graph, estimate the parameters and finally use the residuals to recover the atmospheric phase delay. The PS candidates in the urban area of La Mariscal, correspond to the constructions of the existing buildings which remain stable over time both in radiometry and in the interferometric phase [24]. The image stack used was the most important factor for the pixel coherence estimation, since it allowed identifying suitable PS for the ground displacement analysis. Insufficient use of images will produce an overestimation of coherence throughout the scene, resulting in an overestimation of the PS, therefore, in false displacements. PS identification is generally considered reliable when 20 or more images are used. Estimate APS: PS-InSAR processing is affected by different atmospheric conditions at the acquisition time. APS atmospheric noise reduction is important as it improves the response signal in coherence and phase of the images to obtain more accurate terrain displacement data. It is estimated using spatial temporal filters. The estimated APS was removed from the results. The remaining phases are used to estimate the topographic height error and linear deformation velocity.

*Procedure 5: Multitemporal Analisys (E).* From the previous atmospheric noise reduction procedure, the radar interferometry multi-temporary interferometry analysis was applied: MT-InSAR for persistent scatters (PS). This procedure allowed the identification of dispersers whose signal is dominant within the total dispersion of the observed cell. This is the last process in the generation of the deformation map. It consists of an estimation of the strain rate from the time series obtained. The time series deformation maps obtained are made up of thousands of PS. Each PS is associated with the value of the annual linear velocity (mm/year), estimated during the analyzed period and the accumulated displacement on each acquisition date of the sensor. Finally, to ensure the results of height, velocity and accumulated displacement, the process to reduce atmospheric noise that can cause false signals and interpretations is quickly detailed. This noise was reduced by applying the APS estimation tool.

A prerequisite in the analysis with PS-INSAR is that the signals along time series of SAR images must remain consistent for the extraction of PS points and analyze their dispersion. In this case, to measure the relative displacement and accumulated displacement based on a reference point, a stable point (an artificial construction anchored to the ground, for example) was selected whose peak in the histogram has a residual height of value 0 indicating that this reference point is on the ground (points that are not on the ground are more likely to be unstable).

PS-InSAR measures the movement relative to this reference point, therefore it is important to select a point that is more stable as a reference point.

The program chooses the Persistent Dispersion Candidates (PSC) points based on their location in a connection network based on a threshold value with coherence greater than >0.8. These points are generally parts of artificial civil structures to analyze the dispersion of amplitude stability around those points, drawing a coherent point connection network (Delaunay’s spatial connection graph). This procedure estimated the coherence of the connections in the parameters analyzed at each point of the network with a linear trend such as height and travel speed. Finally, a high temporal coherence of the connections of points in the network was obtained.

After estimating the previous parameters with high coherence, the atmospheric noise was removed. The analysis performed in this article performs the INSAR analysis under the concept of estimating the APS to reduce atmospheric noise and time series multitemporal analysis, with a stack of 32 images (2015–2019) that allowed to determine the movement of the topographic surface according to the variation of the phase for each pixel in time. The number of images in the stack (32 images) were chosen which showed a coherence greater than or equal to 0.65. See Figure 4C. The APS atmospheric noise estimation with high coherence for the whole set of points through the use of inverted residuals (IR) and the analysis of noise from other sources was processed with a non-linear spatial distribution (few points, points too dispersed or orbital inaccuracies), ensuring that the final coherence is satisfactory.

The integrated velocity and integrated accumulated displacement were calculated taking into account that the accumulated displacement = speed × time. At the reference point, the peak of the integrated velocity histogram and integrated cumulative displacement is tending to zero. This means that most points have zero relative velocity when approached and compared with the reference point.

**Figure 5:**
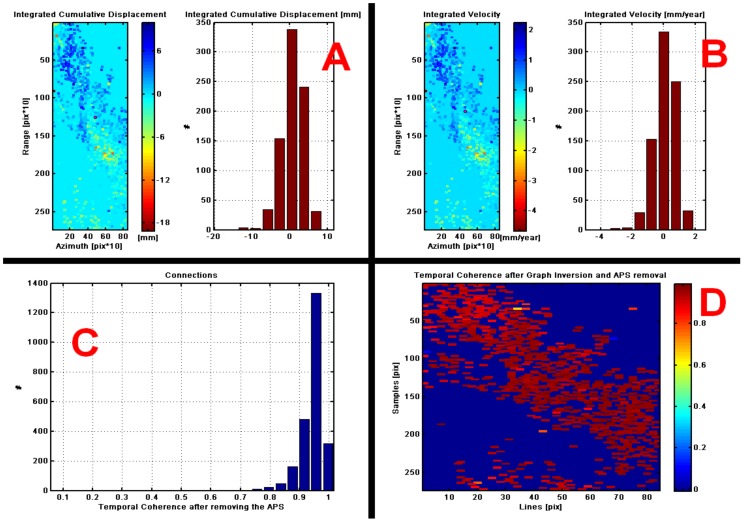
(**A**)The plot of integrated cumulative displacement histogram of connections, (**B**) Integrated velocity, (**C**) Histogram and (**D**) Temporal coherence after APS remove.

The histograms helped to verify that the connection velocity and the residual height of the connection, in checking the distribution of the residual velocity/height value, are consistent as seen in the Figure 5A–C, where a histogram is seen without jumps and high coherence (connection lines with a tendency to red), Figure 5D.

**Figure 6:**
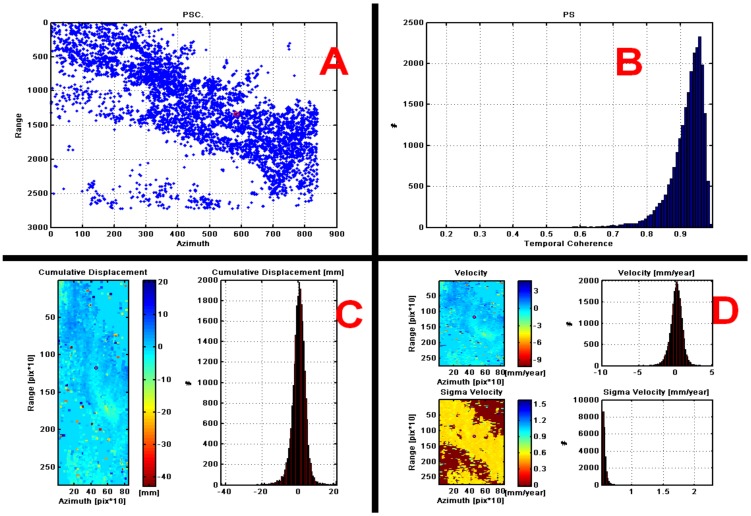
(**A**) 5,277 points for multi-time analisys without atmospheric noise. (**B**) Histogram of scattered points to check the multitemporal analysis of coherence for the estimation of accumulated displacement (**C**) and velocity (**D**).

Subsequently, a multi-time analysis without atmospheric noise was performed based on the “amplitude stability index” and from a threshold from which a set of points of the connection network was chosen to calculate the time series (5277 points) (Figure 6A,B).

The height and the linear trend were estimated between probable ranges of [−20, 60] and [−20, 60] respectively, to analyze the temporal consistency selected. In this case, the consistency was high, so the estimation of the estimated parameters (cumulative displacement, velocity) improved (Figure 6C,D).

**Figure 7:**
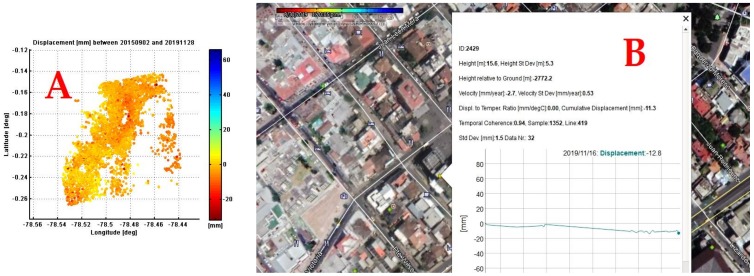
(**A**) Scatter plots of velocity for each point of the area. (**B**) Multi-time displacement and velocity analysis (2015–2019 series).

Dispersion diagrams were used to verify the results that were previously processed based on the coherence and stability of the points dispersed in the time network calculated in relation to a threshold value of 0.8, obtaining velocity dispersion diagrams for each point in the area, Figure 7A and the displacement and speed in a series of time between 2015–2019, Figure 7B.

### 2.2. Phase 2: Analysis of the Space Syntax (Brief Review of the Method)

Space syntax is a set of techniques to analyze spatial designs and patterns of human activity behavior in buildings and urban areas. It is based on the idea that all spaces in an urban environment are interconnected and that their parts are linked together. This geographical approach was created as a tool to help architects simulate the possible social effects of their designs, by Hillier, Hanson and others in the late 70s and early 80s of the last century [25,26].

With this theory, human spatial behavior in urban environments can be predicted and urban morphology (the shape of cities) can be analyzed. In addition, the spaces can be broken down into components, which are analyzed as networks of choice in maps and graphs that describe the relationship of integrity and connectivity of those spaces [27]. The space syntax in this study has been applied based on two fundamental components [28]:

(1) Space representations: Spatial elements are represented through their geometric shapes and how people experience them. They can be derived geometrically (for example, point, axial line, segment, convex and isoview space) or defined functionally (for example, rooms in the building). Axial line, straight line (the longest straight line that represents the maximum extent of a spatial point. This axial line can chain two polygons or convex spaces). Segment (it is the section of the axial line or street or road that extends between two intersections). Isoview, visibility polygon or visible space (it is a set of all visible points from a given point of view in space with respect to an environment. The shape and size of an isoview can change with position). Convex space or polygon (is that space in which no straight line drawn between two points). Axial lines and convex polygons are known as axial maps or convex maps.
(2) Spatial relationships analysis: The relationships between spatial elements result from its configuration. These relationships can be objectively analyzed using various measures, including integration, choice and depth. These measures reflect the two fundamental elements in the human movement: first, the selection of a destination and, second, the selection of a route. One measures the ease of access (integration) and the other measures the step flow (choice).

Integration (measures how many turns must be done from one segment of the street to reach all other segments of the street in the network, using the shortest routes. If you analyze the number of turns required to reach all segments in the graph, it is said that the analysis measures the integration in the radius ‘n’), the choice (which represents the number of intersections that must be crossed to reach a street), and depth distance (the linear distance from the central point of each street segment to the central points of all other segments).

In this study, space syntax was used to know where people are, how they move, concentrate and relate to each other, depending on the urban structure and land use. With these concepts, based on the intensity of the seismic exposure (accumulated displacement with INSAR), the transit-concentration of the population and the level of ease in the displacement from a vulnerable to a non-vulnerable space by safe routes were quantified.

**Figure 8:**
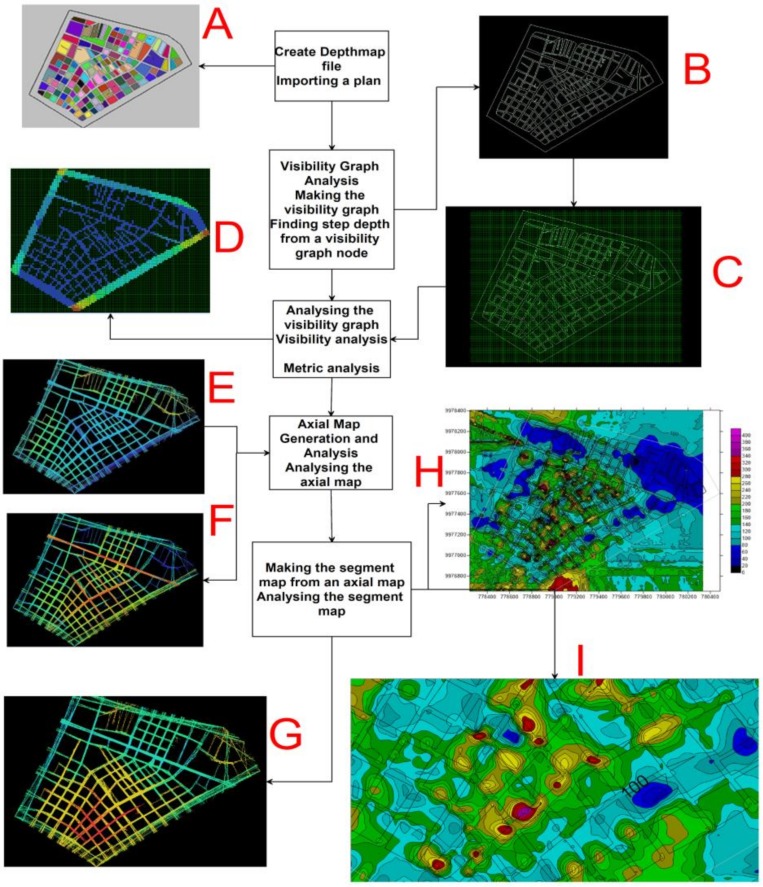
Flow process diagram with DepthmapX used to analyze the spatial relationships of the basic spatial elements, according to the theory of Space Syntax. The description of each process (**A**–**I**) is described in the text.

To analyze the relationships of the basic spatial elements, the open source DepthmapX [29,30] program has been used. The objective of the software is to produce a map of open space elements, connect them through some relationship (for example, intervisibility or overlay) and then perform a graphical analysis of the resulting network. In Figure 8 can see the process flow used for spatial analysis with DepthmapX.

The process begins with the definition of population movement and land occupation, as the fundamental functions of an urban design, where the possibility of population transit (permeability) in all areas is the priority condition for a functional design structure [31].

A representation of the spatial structure of La Mariscal (A) has been drawn on a convex map of 2D houses and the structure of the open space of the building blocks (B). An axial map can be the minimum set of lines that pass through each convex space, lines that form the axial links. The urban space (A) can be represented by the set of smaller, longer and more passable axial lines. These axial lines are represented by a graph (C).

The different connectivity values (degrees) for each vertex are highlighted. The vertices that have more connections with their immediate neighbors will have greater connectivity (colors with a tendency towards red) (D), these connectivity values are illuminated on the axial map to reveal the local network structure of the street spaces (E). To make the axial map of a block, DepthmapX first found the longest straight line that can be drawn in continuous space. Then, the second longest line continues to draw all the lines that can be joined without repeating them [32]. This process was developed in the following depth map based on the topographic map in which the blocks and streets of the sector have been delimited. In a following process (D) based on the 2D topographic plane, the visualization integration graph was prepared, for which in DepthmapX a grid was activated in order to get the locations of visibility points from which this graph was drawn and finally the VGA visualization graphic within the boundaries of the neighborhood was analyzed, with display radios between 200 m, 400 m, 800 m and radius “n” that takes into account the entire urban network (D).

Accessibility to buildings is carried out through streets and open spaces, which are indicators to know how people move around these architectural elements. These connectivity values are then illuminated (tendency towards red) in the axial map to reveal the structure of the local network and the relationship between streets and spaces (G.), which are generally the ones that show the greatest movement of people and pedestrian density. Finally, the intensity maps of visualization and pedestrian flux-density were transformed to raster-grid (I) formats, to later integrate them with the accumulated displacement maps obtained with PSI-INSAR to predict the exposed of buildings where social activities are developed and daily economic conditions that may be affected by a major earthquake [33].

In this analysis, open spaces (parks, greenways, reserves, etc.) were integrated, including public open spaces (streets and squares) and private open spaces (gardens, playgrounds) [34]. In the event of an extreme earthquake, these open areas become a refuge for victims and a temporary home for thousands of people who need to adapt quickly to their new environment for days, months or even years after the earthquake.

This proposal also focuses on “the projection of the city after the earthquake”, which is defined as a projection of the urban structural system that may be affected by its partial or total destruction and the creation of non-accessible areas (fractures in civil structures, subsidence and collapses) [35,36].

## 3. Results

**Figure 9:**
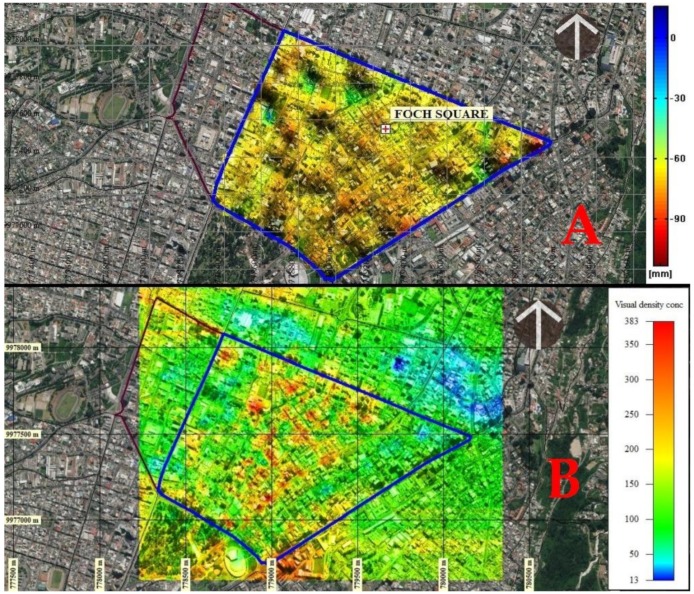
(**A**) Results of cumulative displacement obtained from INSAR, 2015–2019 period. In (**B**) Visual integration in the sector. The colors towards the red represent ground lift and in blue subsidence on the accumulated displacement map. Colors with a tendency towards red represent greater visibility on the vsisibiity intensity map. Graphical scale 1,10,000.

The results of the accumulated displacement obtained from PS-INSAR in millimeters for the period 2 September 2015 to 28 November 2019, can be seen in Figure 9A and has a total symmetrical process range between −20 to 60 mm. The adjusted data of the multitemporal series show a range of movement in the La Mariscal area between 4 and 15 mm per year, a range compatible with the system of blind geological faults sections of the Quito fault with NS orientation, which has an estimated maximum lifting rate of 0.8 mm/year [37].

In (B) the visual integration from various points of view within the area of study is presented. In both cases the red colors represent the greatest accumulated displacement and greater visibility that implies greater population flow and concentration.

The integration of the processes (I) in the flowcharts of the two methodologies used simple map algebra by intersection, addition and multiplication of interpolated files on the same annual accumulated displacement scale and population density visualization concentration. This process was performed in QGis [38,39,40].

**Figure 10:**
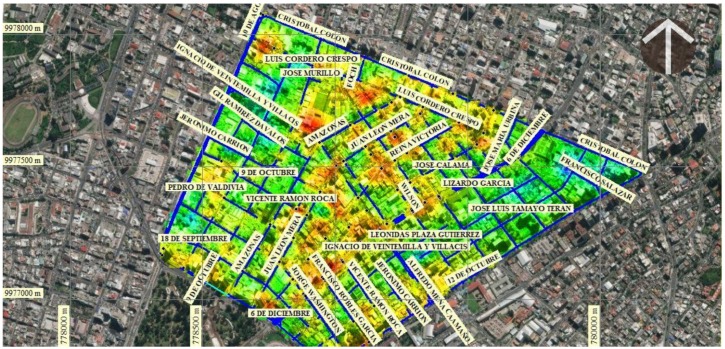
The integration of the information obtained by PS-INSAR and the Analysis of Spatial Syntax determined the existence of several constructed areas exposed to intense deformation-speed of displacement of the land very susceptible to an earthquake greater than 6–7 Mw (colors with tendency to red).

This integration of results determined that the areas are exposed to deformations of high to very high intensity due to the accumulated displacement of the soil and the intense density of population concentration (colors with a tendency to red) that can possibly be destroyed by an earthquake. These areas correspond to the central part of the neighborhood with 59.712% of total buildings and segments of vulnerable streets from Avenida Colón in the north to Avenida Patria to the south and from Avenida 12 de Octubre to the east, to the 10 de Agosto Avenue to the west. The concentration of traffic, density and flow of pedestrians are generally found in this area and in the upper northwest corner there is an area of very high exposure on Luis Cordero Crespo Street and south on 6 de Diciembre Avenue (Figure 10).

The exposure range value (low, medium, high and very high) was compared with the study of vulnerability of seismic risk for residential structures located in the urban parish of La Mariscal, prepared by Parra et al. [9]. According to this study, the largest number of buildings that would reach or exceed extensive structural damage is in the range of 27.8 to 39.1%, and is in the La Mariscal area. This study was based on the identification of constructive typologies in the area to which the author assigned a type of vulnerability differentiated by its constructive structure, in addition to a characterization of the seismic hazard scenario, identification of load capacity curves, mainly of typologies constructive Finally, the author of that study calculated the structural and non-structural damages of the buildings, using the fragility curves corresponding to each typology in a HAZUS classification [7] with the following degrees: none, mild, moderate, extensive and integral.

**Figure 11:**
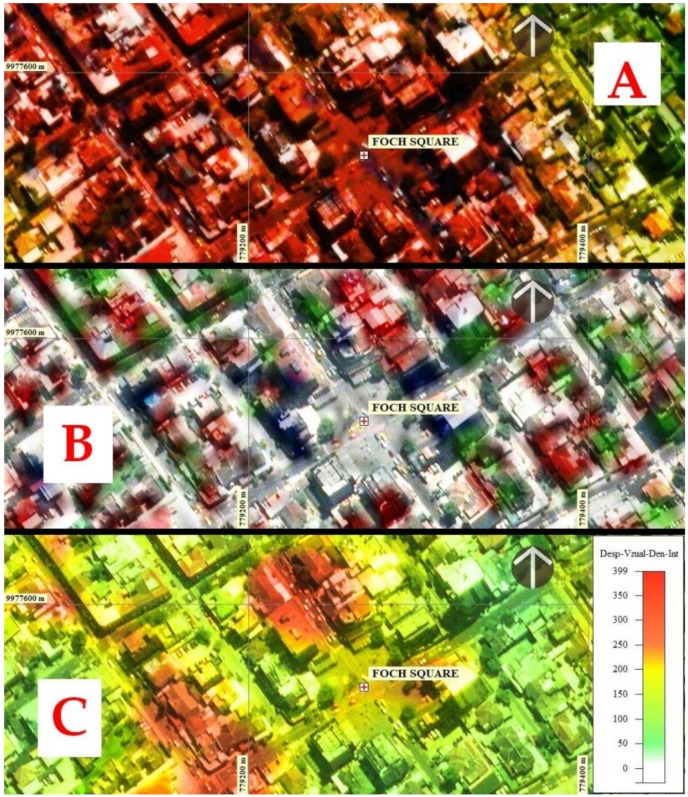
Concordance between the methods applied to determine exposure-vulnerability in Foch Square, considering INSAR-space syntax and the seismic risk study prepared by Parra [9]. (**A**) Foch Square and its surroundings. (**B**) Very high to high seismic vulnerability. (**C**) Busiest places in the sector.

This comparison determined that the exposure analysis of the population concentration-instructions, considering the PSI-INSAR methodology-Analysis of spatial syntax and the study of Seismic Risk carried out by this author, are complementary since there are coincidences between the parameters of the maximum population concentration of accumulated displacements proposed in this study with the degrees of seismic vulnerability obtained in the mentioned study. This coincidence can be seen in Figure 11 in which (A) shows the sector of Foch Square and its surroundings, which are the sectors with the largest agglomeration of economic-productive activities represented in red and where the seismic model located areas of Very high to high vulnerability (B), coinciding with the results obtained in this proposal (C).

There is a high degree of coincidence between the two methodologies in which the intensity of the degree of moderate seismic vulnerability (green color) to complete (red), coincides with the degree of exposure of the average (yellow-orange) to very high (color with a tendency to red) presented in this article.

**Figure 12:**
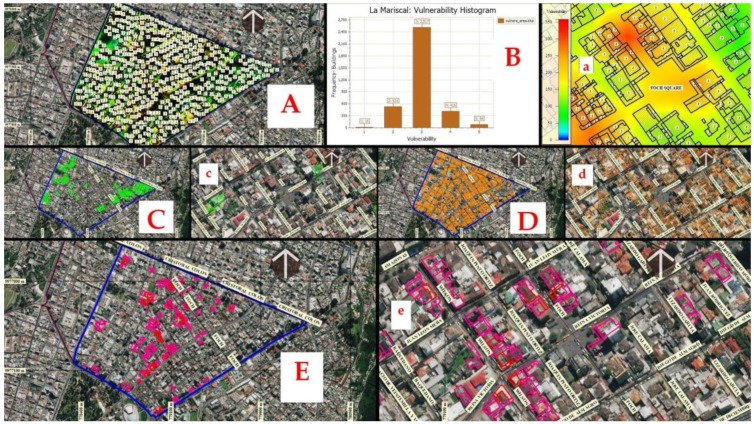
Buildings exposure based on accumulated displacement for the 2015–2019 period and density of population concentration. (**A**) Degree of exposure of buildings based on accumulated displacement. (**B**) Standardized exposure intensity histogram. (**C**) Foch Square, the busiest place in the sector. (**D**) Buildings with medium exposure. (**E**) Buildings with high and very high exposure. In (a, c, d, e) Foch Square, the busiest place in the sector.

In Figure 12A, the degree of exposure of buildings has been represented based on the accumulated displacement in mm for the 2015–2019 period and the visual concentration and population density. Areas exposed to high to very high exposure are represented with a color that tends to red, areas of medium exposure with colors to yellow and areas of low-no exposure has been represented with colors that tend to green-blue respectively. Figure 12B shows the histogram of the standardized exposure intensity of buildings where 1 is none, 2 low, 3 medium, 4 high and 5 very high. The histogram represents the frequency of buildings against exposure.

Figure 12C shows 16 buildings without exposure and 531 buildings with low exposure. In Figure 12D, 2517 buildings with medium exposure were represented. In Figure 12E, 416 buildings with high exposure and 85 buildings with very high exposure can be seen. According to the exposure analysis of the constructions of the sector of Figure 8, the exposure of medium to very high degrees implies that in case of an earthquake of more than 6–7 Mw, the streets of the neighborhood can be partially or totally destroyed. Depending on the intensity of exposure of the streets of the sector, the best known and least exposed routes to be destroyed that the population uses daily have been located and will serve as safe escape routes to reach low exposure areas (green-blue safe areas). In (a,c,d,e) Foch Square images, the busiest places in the sector.

**Figure 13:**
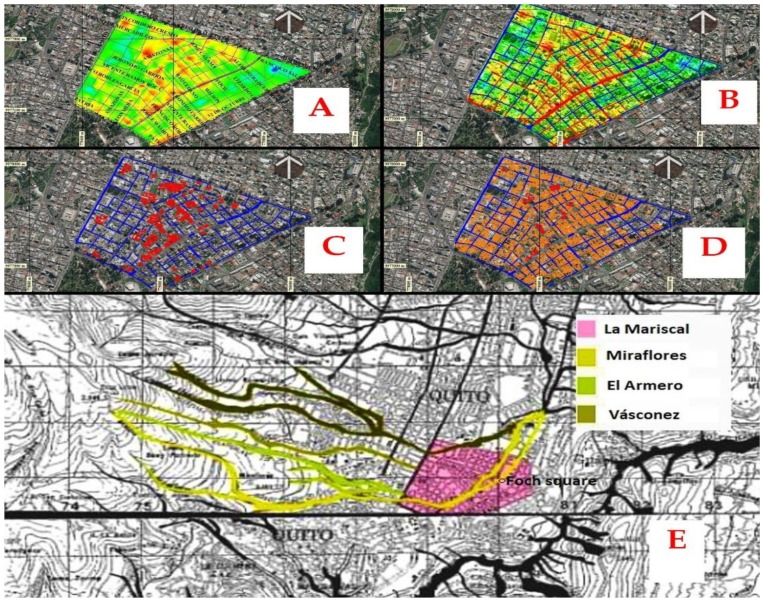
(**A**) All exposure of street. (**B**) Exposure streets with red and non-exposure streets in blue. (**C**) Street vectors with high to very high exposed. (**D**) Streets vectors of medium, high and very high exposure. (**E**) The full ravines and the position of the Foch square. The image is of low resolution from its origin [18].

The exposure of the streets in general is seen in Figure 13A All streets, exposed and unexposed. In Figure 13B the streets exposed and capable of being destroyed before a major earthquake are shown in red with a length of 19.42 km corresponding to 67.6% of the total streets and those not exposed with 15.41 km of blue, with 32.4% of the total of streets. In (C), the vectors of the most exposed streets are seen with intensities from high to very high. In (D) the vectors of the streets of medium, high and very high exposure are seen. The most exposed construction areas are within walking distance in some cases, such as in the sector between the intermediate streets of areas without exposure. The central part of La Mariscal and Plaza Foch are in the path of the Miraflores filled ravine and possibly the accumulated displacement obtained with PSI-INSAR is the result of the differential collapse of this filling. This landfill affects the entire sector, placing Plaza Foch in one at a very high degree of exposure, especially on one of the flanks of the creek (E) [18].

## 4. Discussion

The proposed methodology to develop the exhibition map of the buildings in the neighborhood of La Mariscal, based on the accumulated displacement calculated with PS-INSAR and the spatial syntax (intensity of visual concentration-population density) is an innovative alternative complementary to the traditional seismological methods to improve the prognosis of the seismic threat and can serve as an early warning system in the event of an earthquake greater than 6–7 Mw.

The accumulated displacements, plus the intensity of visualization-density of population in raster format, were integrated into maps of exposed of the buildings in the same scale of work without having problems of geometric spatial corrections.

The detail obtained from exposed buildings is very different from the maps that can be obtained with methodologies that use traditional cartographic methods, due to the spatial resolution of the images, of the digital elevation model that in this case is 5 m and the equations of governance of the processes belonging to the employed programs.

The proposed methodology used by PS-INSAR and space syntax is a technique that provides a new vision of what happened and can happen with respect to the exposure of buildings and the population found in tectonically deformed areas and in landfills of filled ravines internal.

The parameters include displacements and the concentration of the population in amusement centers and public services in these filled ravines and can quickly determine and prepare forecast maps, creating alerts that will serve the authorities to take structural solutions to reduce these hazards.

The possible evacuation routes in some cases are surrounded by other exposed routes, so other alternatives have been chosen, such as evacuation routes that are not associated with the movement of the population’s daily life patterns. The most exposed construction areas are within reach of safe escape routes and in sectors between the intermediate streets of unexposed areas. The location of safe escape routes depends on the intensity of the high and very high exposed areas in the study area.

**Figure 14:**
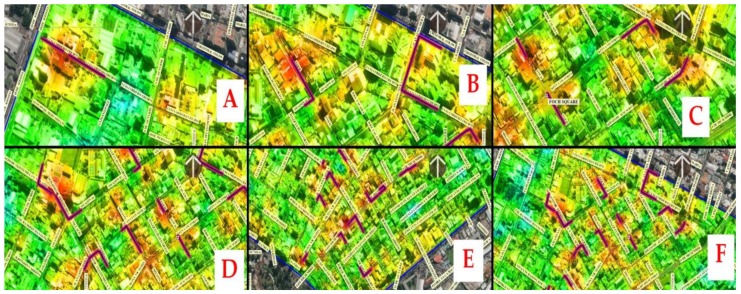
Escape routes near the areas of greatest exposure that the population can reach in a very short time between 1 and 4 min (red-black double line routes in the figures from **A**–**F**).

In general, escape routes are close to the areas of greatest exposure that the population can reach in a very short time between 1 and 4 min. These times have been calculated by analyzing the speed of a person/distance traveled in QGis (complement of the road map), for which the distances are measured to reach all segments of the designated safe streets of the network from an exposed area with a moderate speed of 2 m/s. These red-black double line routes can be seen in the sequence of A–F images in Figure 14.

## 5. Conclusions

The ease of unifying these two concepts of PS-INSAR analysis and spatial syntax allowed previously determining the exposure of the urban space network of La Mariscal in any of its parts, due to the occurrence of an earthquake greater than 6–7 or Mw.

The neighborhood of La Mariscal has areas with high to very high exposure, which represent 60% of its total area due to the accumulated displacement of the land and the population concentration flow. This implies that the population that travels and attends entertainment, recreation and public services centers are in very high exposure to a major earthquake between 6–7 Mw as those that have occurred historically.

The exposed polygon goes from Avenida Colón, on the northern edge, to Patria Avenue, on the southern edge and from 12 de Octubre Avenue, on the eastern edge, to 10 de Agosto Avenue on the western edge. After the analysis to identify segments of streets that have difficulty being used as safe evacuation routes (due to their high to very high exposure) was completed, streets have been identified that can be used as valid safe routes for the evacuation of the population to safe areas

The use of free information, such as Sentinel 1A images, digital elevation models and free software for spatial syntax analysis, such as DepthmapX, is a benefit for the investigation of this and other types of natural threats, such as mass movements and the floods.

The proposed methodology allowed us to obtain safe escape routes that can take the population to safe areas in case of an earthquake of more than 6–7 Mw. A beneficial coincidence was the concordance of the responses between the proposed methodology and the seismic model.

Coincidences that served to improve the interpretation of the exposure of the buildings and streets of the studied area, in case of an earthquake greater than 6–7 Mw.

This methodology can be proposed to the municipality of the city of Quito and to the municipalities of other provinces to reduce evacuation times and simplify the process of searching for safe routes. In addition, to improve response processes after the generation of a large earthquake, this is associated with the effective response in the evacuation process.

Although the methodology used and its potential to determine the deformations of the ground and the accumulated travel speed of PS-InSAR is very objective, there are several limitations that can determine erroneous results in results related mainly to technology due to the impossibility of reducing the signals false. Changes in the reflectivity of the ground cause radar de-correlation, which makes the InSAR phase illegible. In addition, atmospheric water vapor delays radar signals, making phase readings inaccurate. As for the spatial syntax methodology, there are no limitations for its use.
